# Comprehensive analysis of metabolites produced by co-cultivation of *Bifidobacterium breve* MCC1274 with human iPS-derived intestinal epithelial cells

**DOI:** 10.3389/fmicb.2023.1155438

**Published:** 2023-04-13

**Authors:** Akira Sen, Tatsuki Nishimura, Shin Yoshimoto, Keisuke Yoshida, Aina Gotoh, Toshihiko Katoh, Yasuko Yoneda, Toyoyuki Hashimoto, Jin-Zhong Xiao, Takane Katayama, Toshitaka Odamaki

**Affiliations:** ^1^Next Generation Science Institute, Morinaga Milk Industry Co., Ltd., Kanagawa, Japan; ^2^Division of Integrated Life Science, Graduate School of Biostudies, Kyoto University, Kyoto, Japan; ^3^Technology Research Laboratory, Shimadzu Corp., Kyoto, Japan

**Keywords:** *Bifidobacterium*, intestinal epithelial cells, metabolite, co-culture, host–microbe interaction, indole-3-lactic acid

## Abstract

Examining how host cells affect metabolic behaviors of probiotics is pivotal to better understand the mechanisms underlying the probiotic efficacy *in vivo*. However, studies to elucidate the interaction between probiotics and host cells, such as intestinal epithelial cells, remain limited. Therefore, in this study, we performed a comprehensive metabolome analysis of a co-culture containing *Bifidobacterium breve* MCC1274 and induced pluripotent stem cells (iPS)-derived small intestinal-like cells. In the co-culture, we observed a significant increase in several amino acid metabolites, including indole-3-lactic acid (ILA) and phenyllactic acid (PLA). In accordance with the metabolic shift, the expression of genes involved in ILA synthesis, such as transaminase and tryptophan synthesis-related genes, was also elevated in *B. breve* MCC1274 cells. ILA production was enhanced in the presence of purines, which were possibly produced by intestinal epithelial cells (IECs). These findings suggest a synergistic action of probiotics and IECs, which may represent a molecular basis of host-probiotic interaction *in vivo*.

## 1. Introduction

Bifidobacteria are one of the major bacterial genera constituting the human gut microbiota, and several strains have been industrially used as probiotics (Turroni et al., 2011; Alessandri et al., 2021). Bifidobacteria produce various beneficial metabolites such as acetic and lactic acids by utilizing hexose sugars (Suzuki et al., 2010). These metabolites contribute to maintaining intestinal barrier function and enhancing immune function (Fukuda et al., 2011; Lee et al., 2018; Morita et al., 2019). Some bifidobacterial species possess a metabolic pathway for synthesizing aromatic lactic acids (ALAs) from dietary aromatic amino acids (Sakurai et al., 2019; Laursen et al., 2021; Sakurai et al., 2021). An example of an aromatic lactic acid is indole-3-lactic acid (ILA), which exerts anti-inflammatory activity *via* the aryl hydrocarbon receptor (AhR) (Ehrlich et al., 2020; Meng et al., 2020). In addition to the above metabolites, certain bifidobacteria produce vitamins and conjugated linoleic acid (Coakley et al., 2003; Sugahara et al., 2015; Mei et al., 2022), which can act as signaling molecules and affect host physiology.

Since probiotics consumed orally exert its effects only transiently in the colon, it remains challenging to investigate the *in vivo* metabolic behavior of bifidobacteria when taken as probiotics due to the complexity of the gut environment, including the interaction of probiotics with host-gut microbes. Thus, an *in vitro* co-culture system capable of mimicking the gut environment can help to determine the metabolites produced by gut microbes in the human intestinal tract. Recently, several culture systems have been developed to address the difference in oxygen demand between the host and bacteria using a static two-layer culture system and microfluidic devices (Shah et al., 2016; Jalili-Firoozinezhad et al., 2019; Shin et al., 2019; Kwon et al., 2022). In addition to these culture techniques, attempts have been made to combine intestinal organoids to create an environment similar to that of the gastrointestinal tract (Sasaki et al., 2020; Gazzaniga et al., 2021; Puschhof et al., 2021; Zhang et al., 2021). Sasaki et al. reported a successful co-culture of colonic organoid-derived monolayers with some anaerobic bacteria, including *Bifidobacterium* species. While these new technologies are still in their infancy, they could advance research on host–bifidobacterial interactions.

Orally supplemented probiotics pass through the small intestine where they interact with intestinal epithelial cells (IECs) and affect the small intestine environment. Some probiotic bifidobacteria were detected in terminal ileum with colony-forming units (Pochart et al., 1992; Takada et al., 2020). Microbes in the small intestine play an important role in host physiology, including metabolism, immunity, and gastrointestinal motility (Saffouri et al., 2019; Kastl et al., 2020; Vonaesch et al., 2022). These reports highlight the importance of understanding the metabolic behavior of probiotics in the small intestine.

In this study, we used a two-chamber co-culture device to evaluate the interaction between *B. breve* MCC1274, which has been reported to improve cognitive function in patients with suspected mild cognitive impairment (Xiao et al., 2020; Asaoka et al., 2022), and induced pluripotent stem cells (iPS)-derived small intestinal-like cells to construct a small intestinal tract-like environment *in vitro*. The aim of this study was to determine how a co-culture of human IECs with the probiotic *B. breve* MCC1274 alters their metabolic behavior.

## 2. Materials and methods

### 2.1. Bacterial culture

*B. breve* MCC1274 was obtained from the Morinaga Culture Collection (Morinaga Milk Industry, Tokyo, Japan). This strain was cultured in De Man, Rogosa, and Sharpe broth (BD Biosciences, Franklin Lakes, NJ, United States) supplemented with 0.05% (w/v) L-cysteine hydrochloride (Kanto Chemical, Tokyo, Japan) for 16 h at 37°C under anaerobic conditions using an Anaero Pack (Mitsubishi Gas Chemical, Tokyo, Japan). The culture medium was centrifuged (1,000 × *g*, 5 min, 4°C), and the pellet was washed with PBS (FUJIFILM Wako Pure Chemical, Osaka, Japan) and suspended to approximately 1.0 × 10^9^ CFU/ml in PBS. The number of colony-forming units (CFU) on transgalactosylated oligosaccharide (TOS) propionate agar (Yakult Pharmaceutical Industry, Tokyo, Japan) was used to calculate the number of *B. breve* MCC1274 cells.

### 2.2. Monolayer culture of human small intestinal-like epithelial cells

Monolayers of human iPS-derived small intestinal epithelial-like cells were generated from FUJIFILM human iPS cell-derived Small Intestinal Epithelial-like Cells (F-hiSIECs) (FUJIFILM Wako Pure Chemical, Osaka, Japan) according to the manufacturer instructions with minor modifications. Briefly, Transwell culture inserts (12-well insert, 0.4-μm pore polyester membrane; Greiner bio-one, Kremsmunster, Austria) were coated with Matrigel (Corning, Corning, NY, United States) diluted 1:30 in DMEM/F12 medium (Gibco, Thermo-Fisher Scientific, Waltham, MA, United States) at least 1 day before F-hiSIEC seeding. Next, thawed F-hiSIECs were suspended to 1 × 10^6^ cells/mL in seeding medium, and 340 μL of the cell suspension was seeded on each Transwell insert. The F-hiSIEC culture medium was changed every 2–3 days, and in the last medium change before using the co-culture device, antibiotics were omitted from the F-hiSIEC culture medium. Transepithelial electrical resistance (TER) values, measured using the Millicell-ERS system (Merck Millipore, Burlington, MA, United States), were used to evaluate monolayer maturation.

### 2.3. Co-culture of bacteria and IECs

To co-culture IECs and *B. breve* MCC1274, we used a two-chamber co-culture device (Shimadzu, Kyoto, Japan) (Supplementary Figure S1). The device could culture anaerobic bacteria on the apical side of IECs while maintaining aerobic conditions on the basolateral side by sealing the culture cup with the Transwell culture insert. IECs monolayers on Transwell inserts were placed on the device, and F-hiSIEC culture medium without antibiotics was filled up at the basolateral side of the device. The device was then transferred into a TYPE-B, flexible vinyl anaerobic chamber (Coy Laboratory Products, Grass Lake, MI, USA). On the apical side, the medium was replaced with Yeast Casitone (YC) medium and left in the anaerobic chamber overnight. The composition of the YC medium is described in the Supplementary Table S1. The device was maintained at 37°C for 24 h before starting co-culture. Next, 1% (v/v) of a pre-conditioned *B. breve* MCC1274 suspension was inoculated into the apical medium. As the control group, the same volume of bacterial suspensions was added to conical tubes containing an equal volume of YC medium on the apical side of the device but no IECs. After incubating for 24 h at 37°C, the apical medium of the device and the culture medium of the conical tube were collected to calculate the number of *B. breve* MCC1274 by counting the number of CFU on TOS propionate agar (Yakult Pharmaceutical Industry). Then, the culture medium was centrifuged (10,000 × g, 5 min, 4°C) and the supernatant was stored at −20°C until use.

### 2.4. Culturing *Bifidobacterium breve* MCC1274 with purines

*B. breve* MCC1274 was cultured in YC medium supplemented with 200 μM each of adenosine, inosine, hypoxanthine, and xanthine (FUJIFILM Wako Pure Chemical) for 24 h under anaerobic conditions. Then, the culture medium was centrifuged (10,000 × *g*, 5 min, 4°C), and the supernatant was stored at −20°C until analysis.

### 2.5. Metabolome analysis

A comprehensive hydrophilic metabolite analysis was conducted using the ω-Scan package (Human Metabolome Technologies (HMT), Yamagata, Japan). Briefly, culture supernatant samples were diluted with 10 μM internal standard sample, and centrifuged (9,100 × *g*, 4°C, 60 min) using a centrifugal filter (5-kDa Ultra-free MC-PLHCC, HMT). Next, capillary electrophoresis Fourier-transform mass spectrometry (CE-FTMS) was used for metabolomic analysis of the samples. Peaks with a signal-to-noise ratio (S/N) of three or higher were extracted using the MasterHands software (v 2.19.0.2 Keio University, Yamagata, Japan). Peak information, such as mass-to-charge ratio (m/z), peak area, and migration time (MT), was also obtained using the same software. The extracted peaks were annotated based on the m/z values and MT, using the HMT metabolome database. Three samples from one experiment were analyzed in this analysis. Metabolites with initials XC or XA, which were detected in multiple biological sample types in the HMT assay results, were annotated by the HMT Known-Unknown peaks library. The relative peak area were shown in Supplementary Table S2.

### 2.6. Quantification of metabolite concentrations using LC–MS/MS

The concentrations of the aromatic amino acids and aromatic pyruvic acids in the medium were quantified using liquid chromatography–tandem mass spectrometry (LC–MS/MS) using a Vanquish HPLC connected with the TSQ-FORTIS (Thermo Fisher Scientific, Waltham, MA, United States). For deproteinization, culture medium supernatant was mixed with methanol at a ratio of 1:9 and centrifuged (10,000 × g, 5 min, 4°C) to remove aggregation. The supernatants were evaporated (miVac Quattro LV, Genevac Ltd., Ipswich, United Kingdom) and resuspended in Mobile phase A with 100 ng/ml 1-methyl-2-oxidole as the internal standard. As an elution method, method 1 described below was used in the experiments for comparing co-culture and monoculture supernatant, while method 2 for evaluating the effect of purine supplementation. The conditions used to analyze in each elution method are described in Supplementary Tables S3, S4.

#### 2.6.1. Method 1

LC–MS/MS quantification was measured using an XBridge® C18 column (4.6 mm × 150 mm, 5 μm) (Waters Corporation, Milford, MA, United States) and the protocols mobile phase A (1 g/L ammonium acetate) and mobile phase B (methanol), were applied at a flow rate of 0.2 mL/min. Gradient elution was performed by changing the percentage of mobile phase B in the following steps: (i) maintenance of 2% for 2 min, (ii) increase from 2 to 35% until 5 min, (iii) increase from 35 to 75% until 21 min, (iv) increase from 75 to 77% until 25 min, (v) increase from 77 to 99% until 30 min, (vi) maintenance of 99% until 38 min, (vii) decrease from 99 to 2% until 40 min, and (viii) maintenance of 2% until 55 min. Six samples from two independent experiments were analyzed in this assay.

#### 2.6.2. Method 2

LC–MS/MS quantification was measured using an XBridge® C8 column (4.6 mm × 150 mm, 3.5 μm) (Waters Corporation, Milford, MA, United States) and the protocols mobile phase A (0.5 g/l ammonium formate) and mobile phase B (methanol) were applied at a flow rate of 0.2 ml/min. Gradient elution was performed by changing the percentage of mobile phase B in the following steps: (i) maintenance of 2% for 2 min, (ii) increase from 2 to 65% until 40 min, (iii) increase from 65 to 99% until 45 min, (iv) maintenance of 99% until 55 min, (vii) decrease from 99 to 2% until 60 min, and (viii) maintenance of 2% until 75 min. Three samples from one experiment were analyzed in this assay.

### 2.7. RNA sequencing

The bacterial cells were treated with RNAProtect Bacteria Reagent (Qiagen, Hilden, Germany) and stored at −80°C. The pellets were suspended in TE Buffer (pH 8.0) containing 30 mg/ml lysozyme (Sigma-Aldrich, St. Louis, MO, United States), 5,000 U/ml mutanolysin (Sigma-Aldrich), and 20 mg/ml protease K (Qiagen) and incubated for 1 h at room temperature (20–25°C). Total RNA was extracted using the RNeasy Mini Kit (Qiagen) with an RNase-Free DNase Set (Qiagen). Library preparation was performed using Illumina Stranded Total RNA Prep with Ribo-Zero Plus and IDT® for Illumina® RNA UD Indexes Set A-B Ligation (Illumina, San Diego, CA, United States). The concentration and quality of the extracted RNA and adapter-tagged sequence library were calculated using an Agilent RNA 6000 Nano and Agilent High Sensitivity DNA Kits (Agilent Technologies, Santa Clara, CA, USA), respectively. Sequences were obtained using the NextSeq 1,000 system with the Illumina NextSeq 1000/2000 P2 Reagent kit (100 cycles) (Illumina). Trimming and mapping were conducted using CLC workbench (v 8, Qiagen) software. Normalization from total read counts, and the identification of differentially expressed genes (DEGs) were performed *via* iterative differential expression analysis (DESeq2) using TCC-GUI software (Su et al., 2019). The KEGG (Kyoto Encyclopedia of Genes and Genomes) Ortholog (KO) number and functional annotation of DEGs were assigned using GhostKOALA (v 2.2, Kanehisa et al., 2016). Functional classification and reconstruction of metabolic pathways of annotated DEGs were performed using KEGG Mapper (v 5.0). Five samples monocultured in tubes and six co-cultured samples from two independent experiments were analyzed.

### 2.8. Statistical analysis

To calculate the significance of *B. breve* MCC1274 cell count, the relative abundance of each metabolite in the metabolome analysis, and the concentrations of ALAs and aromatic pyruvic acids, two-sided Welch’s *t*-test was used. The comparison of the effects of purines for ALAs synthesis was assessed using Dunnett’s test. For metabolome analysis, principal component analysis (PCA) and heatmap analyses were performed with the scaled value of the annotated peak intensity. The value of no detection (N.D.) in the metabolomics analysis data was regarded asset at eps (2^−52^). Heatmaps described the significantly different metabolites (*q*-value <0.1), as calculated with the Kruskal–Wallis test and Benjamini–Hochberg post-hoc test. Vertical and horizontal hierarchical clustering was calculated using Spearman’s correlation distance and Ward’s clustering method. For RNA sequencing (RNA-seq) analysis, genes with a *q*-value <0.05 and a fold change (FC) of >2.0 were defined as DEGs. Statistical analyses were performed using R (version 4.1.3) with the FactoMineR package (version 2.4) and factoextra package (version 1.0.7) for PCA analysis, the ComplexHeatmap package (version 2.10.0) for heatmap analysis, and the Enhancedvolcano package (version 1.14.0) for visualizing DEGs of RNA-seq data.

## 3. Results

### 3.1. Evaluation of metabolic properties of the basolateral and apical media in the co-culture device

Initially, we confirmed the functionality of the device by evaluating the metabolites in the apical (YC medium) and basolateral sides (F-hiSIEC culture medium) of IECs without co-cultivation with bacteria. Based on total peak-annotated metabolites, we confirmed that distinctly different clusters were formed in the medium of the basolateral and apical sides, as can be seen in PCA (Supplementary Figure S2A) and heatmap analysis (Supplementary Figure S2B). These data indicate that there were less leaks of metabolites from one side to the other, suggesting that IECs were not in a leaky state and this device is suitable for subsequent experiments.

### 3.2. Differences in the metabolic profiles of IEC supernatants in the presence or absence of *Bifidobacterium breve* MCC1274

**Figure 1:**
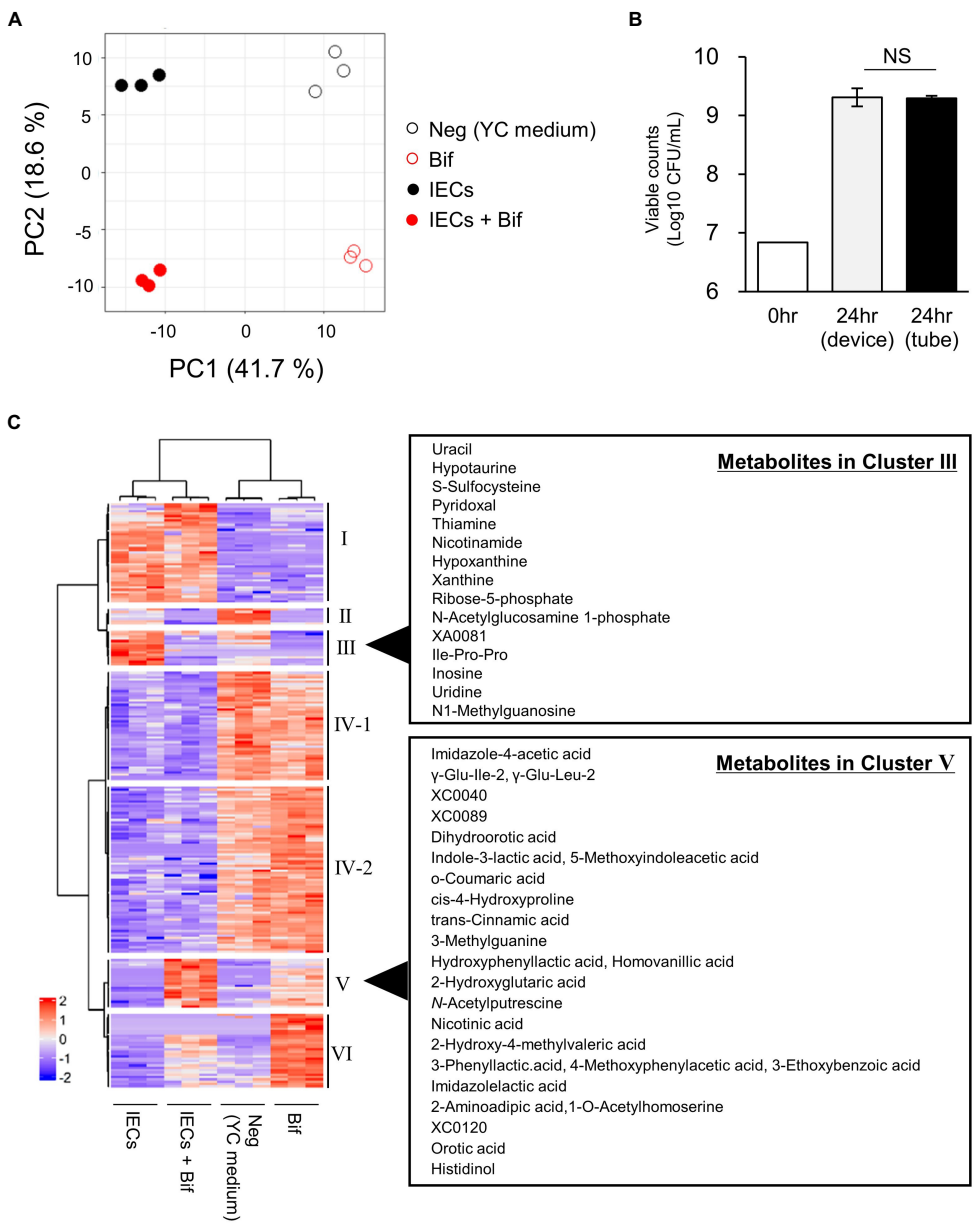
Comparison of the metabolites in the culture supernatants of *Bifidobacterium breve* MCC1274 with or without IECs. **(A)** PCA plot based on the total metabolites detected in the apical side of the device. Neg, YC medium; Bif, supernatant of *B. breve* MCC1274 monocultured for 24 h in tube. IECs, supernatant of IECs monocultured in the apical side of the co-culture device cultured for 48 h; IECs + Bif, supernatant of IECs co-cultured with *B. breve* MCC1274 in the apical side of the co-culture device cultured for 24 h. **(B)** Viable cell count of *B. breve* MCC1274 before (0 h) and after (24 h) cultivation in the tube as a monoculture, and in the device as a co-culture with IECs. The data represent the average of two samples (0 h) and six samples (24 h) from each group, from two independent experiments (Average ± standard deviation). Standard deviation were not described in before cultivation samples. NS, not significant (*p* = 0.83, Welch’s *t*-test). **(C)** A heatmap of the scaled intensity value of each metabolite with a significant difference (*q*-value <0.1) in the Kruskal–Wallis and Benjamini–Hochberg tests.

To explore the metabolite changes that were caused from the co-cultivation, we compared the metabolites in the supernatants of the probiotic monoculture with those in the apical side of the co-culture device, in the presence or absence of *B. breve* MCC1274. A total of 417 metabolites were detected, of which 237 were significantly different between the groups according to the Kruskal–Wallis test (*q*-value <0.1). The metabolite profile was divided into four distinct groups by PCA (Figure 1A). The groups were separated on the PC 1 axis by the presence of IECs, and on the PC 2 axis by the presence of *B. breve* MCC1274. The number of viable *B. breve* cells was almost the same in both the co-culture and the monoculture (Figure 1B).

To explore the different metabolites in each group, cluster classification based on the relative abundance of each metabolite was performed (Figure 1C; Supplementary Table S2). The metabolites were divided into six clusters as follows:

- Cluster I, the metabolites with higher abundance in the culture medium of IECs.
- Cluster II, the metabolites with higher abundance in the applied medium, but lower in the presence of IECs or *B. breve* MCC1274.
- Cluster III, the metabolites with higher abundance in the culture medium of IECs, but lower in the presence of *B. breve* MCC1274.
- Cluster IV, which is subdivided into two clusters IV-1 and IV-2, the metabolites with higher abundance in the applied medium without IECs.
- Cluster V, the metabolites with higher abundance in the culture medium of *B. breve* MCC1274 with IECs.
- Cluster VI, the metabolites with higher abundance in the culture medium of *B. breve* MCC1274 without IECs.

**Figure 2:**
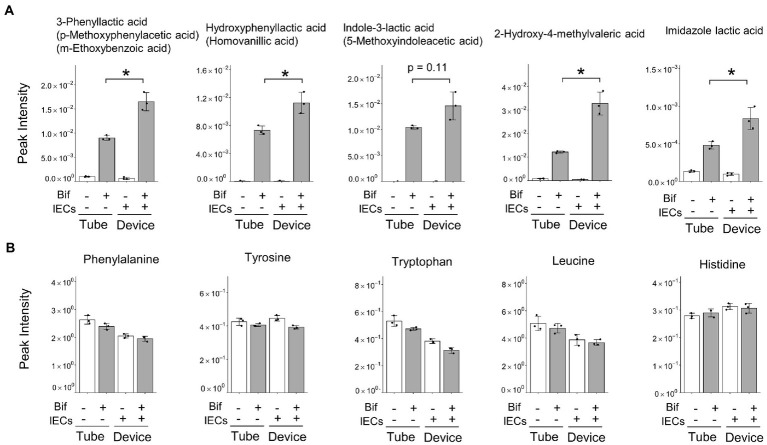
Peak intensity of amino acid-derived metabolites and their precursor amino acids. **(A)** Amino acid-derived metabolites, alternatives are shown in parentheses when peaks were annotated with more than one metabolite. **(B)** Precursor amino acids corresponding to the five metabolites shown in Figure 3. **(A)** Data are expressed as the mean ± SD (*n* = 3),* *p* < 0.05, ** *p* < 0.01 (Welch’s *t*-test).

Cluster V represented the increase in the abundance of metabolites produced by *B. breve* MCC1274 when co-cultured with IECs, compared with that in the monoculture alone. We found that three ALAs, namely, PLA, 4-hydroxyphenyllactic acid (4-OH-PLA), and ILA; as well as the structurally similar metabolites imidazole lactic acid, and 2-Hydroxy-4-methylvaleric acid, fell into Cluster V (Figures 1C, 2A). Some of these ALAs were not annotated as a single substance; for example, a single peak was annotated as both Indole-3-lactic acid and 5-Methoxyindoleacetic acid. In contrast, no significant increase was observed in the amino acids phenylalanine, tyrosine, and tryptophan as substrates for the corresponding metabolite (Figure 2B). These results suggest that the increase in ALAs in the co-culture conditions was attributed to metabolic changes in either *B. breve* MCC1274 or IECs, and not due to a substrate increase in the co-culture environment.

### 3.3. Quantification of ALAs and aromatic pyruvic acids

**Figure 3:**
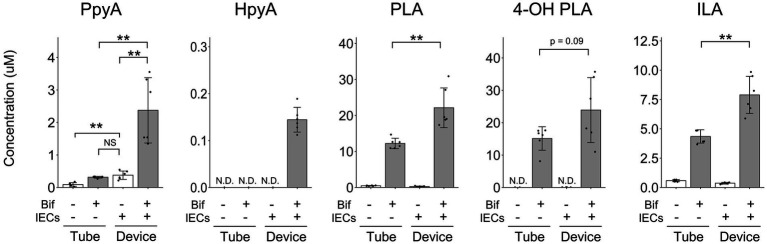
Quantitative analysis of aromatic lactic acids and their precursor metabolites. The concentrations of aromatic lactic acid-related metabolites. The data represent the average of six samples from each group, from two independent experiments. *p < 0.05, **p < 0.01 (Welch’s *t*-test) N.D., not detected.

Since some metabolites shown in Figure 2A were not annotated as a single substance using CE-FTMS, we performed LC–MS/MS to quantify ALAs and the corresponding aromatic pyruvic acids (Figure 3). We confirmed that PLA and ILA concentrations were significantly increased (*p* < 0.01), while 4-OH-PLA concentration showed a tendency to increase (*p* = 0.09) in the co-culture conditions compared with the concentrations in the monoculture of *B. breve* MCC1274. The concentration of the precursor metabolite phenylpyruvic acid (PpyA) significantly increased in the co-culture group (*p* < 0.01), while 4-hydroxyphenylpyruvic acid (HpyA) was only detected in the co-culture group. Contrastingly, the level of indole pyruvic acid (IpyA) was under detection limit (data not shown).

### 3.4. *Bifidobacterium breve* MCC1274 gene expression

**Figure 4:**
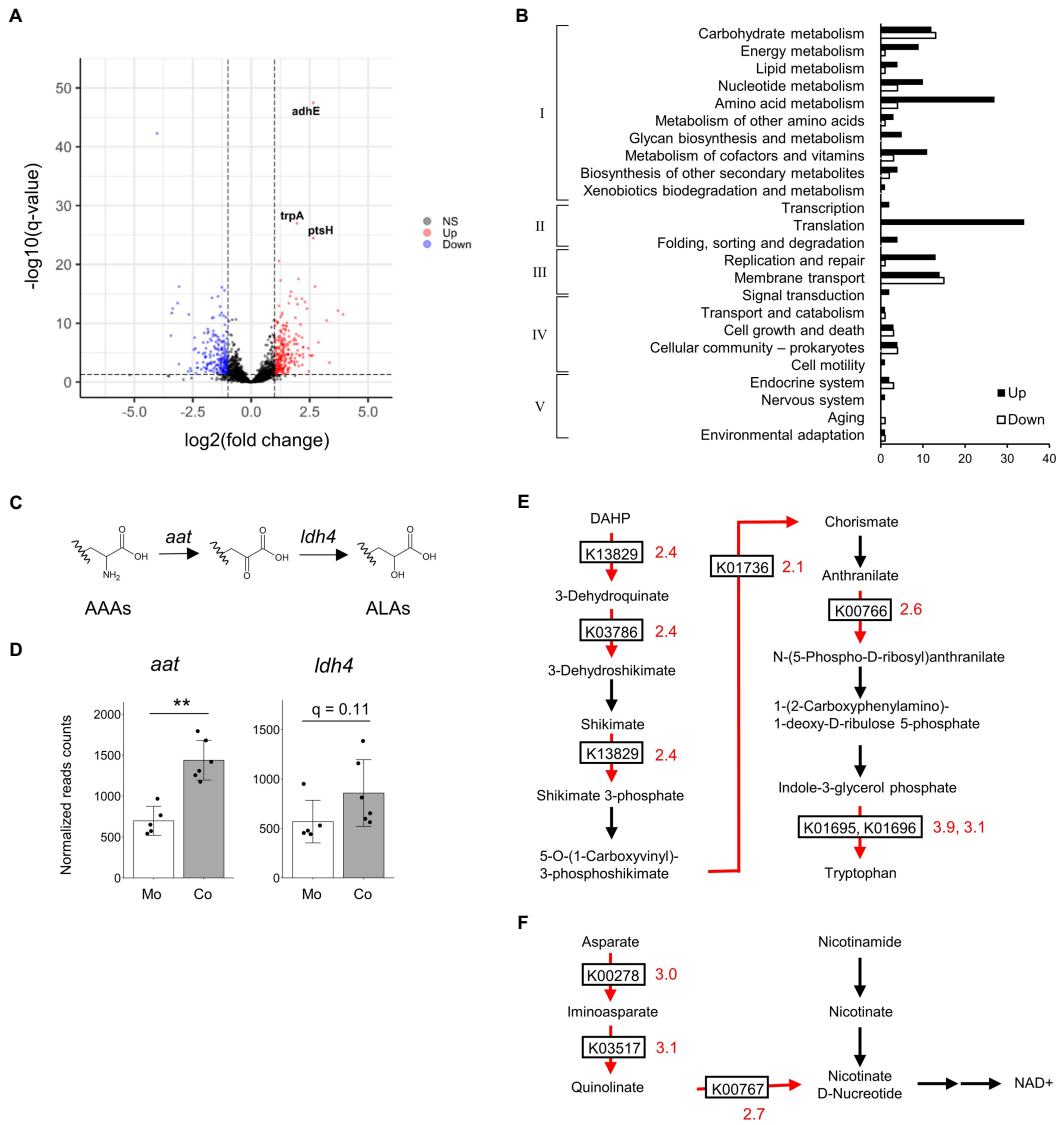
Comparison of gene expression profiles of *B. breve* MCC1274 in monoculture and in co-culture with IECs. **(A)** Volcano plot of total gene expression. Vertical and horizontal dotted lines indicate the fold-change value |FC| = 2 and a *q*-value of 0.05, respectively. Upregulated DEGs in the co-culture conditions are labeled in red, while downregulated DEGs are labeled in blue. RNA-seq was performed on five to six samples from two independent experiments. **(B)** KEGG Orthology (KO) functional classification of annotated DEGs. The horizontal axis represents the number of genes. Categories I–V represent the following mapped KEGG pathway categories: I, Metabolism; II, Genetic Information Processing; III, Environmental Information Processing; IV, Cellular Processes; V, Organismal Systems. **(C)** Aromatic amino acid synthesis pathway. **(D)** The normalized read counts from RNA-seq analysis. Mo, *B. breve* MCC1274 monocultured in the tube; Co, *B. breve* MCC1274 co-cultured with IECs. **q < 0.01 (Deseq2). **(E,F)** Tryptophan synthesis pathway components: **(E)** NADH synthesis **(F)** The red arrow indicates the enzymes whose gene expression was upregulated in co-culture (fold change >2, *q*-value <0.05). Black arrows indicate gene expression that was not significantly changed. The numbers next to the boxes describing KEGG Orthology indicate fold change. AAAs, aromatic amino acids; ALAs, aromatic lactic acids; APAs, aromatic pyruvic acids; DHAP, 2-Dehydro-3-deoxy-D-arabino-heptonate 7-phosphate.

To further clarify the mechanism behind the increase in ALA concentrations, we compared the gene expression profile of *B. breve* MCC1274 in the monoculture with that in the co-culture with IECs. A total of 446 genes were identified as DEGs (Figure 4A). We further classified the annotated DEGs, 173 upregulated and 77 downregulated genes, using the KEGG functional annotation (Figure 4B). The upregulated DEGs were mainly involved in translation and amino acid metabolism pathways. Moreover, the top ten upregulated genes, ranked by *q*-value, included genes involved in amino acid biosynthesis (*trpA, trpBC, livC2*, and *tpiA*) (Table 1), suggesting that amino acid metabolism by *B. breve* MCC1274 increased in the co-culture with IECs.

**Table 1:** List of top 10 up- and down-regulated genes of *B. breve* MCC1274 in the co-culture with IECs compared with the mono-culture.

Rank	KO	Annotation	FC	*q*-value
*Up-regulated gene*				
1	K04072	adhE; acetaldehyde dehydrogenase / alcohol dehydrogenase	6.26	3.0.E-48
2	K01695	trpA; tryptophan synthase alpha chain	3.86	7.8.E-27
3	K02784	ptsH; phosphocarrier protein HPr	6.29	3.0.E-25
4	K01000	mraY; phospho-N-acetylmuramoyl-pentapeptide-transferase	2.27	1.2.E-20
5	K00053	ilvC; ketol-acid reductoisomerase	4.04	5.7.E-18
6	K01925	murD; UDP-N-acetylmuramoylalanine--D-glutamate ligase	2.38	1.2.E-17
7	K03502	umuC; DNA polymerase V	6.61	4.6.E-17
8	K03551	ruvB; holliday junction DNA helicase RuvB	3.24	3.5.E-16
9	K01696	trpB; tryptophan synthase beta chain	3.13	2.2.E-15
10	K01803	TPI, tpiA; triosephosphate isomerase (TIM)	4.48	5.9.E-15
*Down-regulated gene*				
1	–	Hypothetical protein	0.06	2.3.E-43
2	–	Hypothetical protein	0.12	4.2.E-17
3	–	Hypothetical protein	0.42	1.2.E-16
4	–	Hypothetical protein	0.45	6.8.E-16
5	K03484	scrR; LacI family transcriptional regulator, sucrose operon repressor	0.37	3.4.E-15
6	K01768	E4.6.1.1; adenylate cyclase	0.31	6.1.E-15
7	–	Hypothetical protein	0.11	2.8.E-14
8	–	Hypothetical protein	0.40	8.7.E-14
9	–	Hypothetical protein	0.10	2.4.E-13
10	K05349	bglX; beta-glucosidase	0.09	1.6.E-12

FC, Fold change, KO, KEGG Orthology.

Next, we focused on ALA synthesis-related genes. In the co-culture, the gene expression of the aminotransferase gene (*aat*) was significantly increased (2.06-fold, p < 0.01, q < 0.01), while that of the lactate dehydrogenase type 4 gene (ldh4) tended to be increased (1.54 fold, *p* = 0.06, q = 0.11) when compared with the expression of those genes in a monoculture (Figure 4D). Other transcriptional changes related to ALA synthesis in the co-culture included the enhanced expression of genes involved in tryptophan and NADH synthesis (Figures 4E,F).

### 3.5. *Bifidobacterium breve* MCC1274 purine metabolism

**Figure 5:**
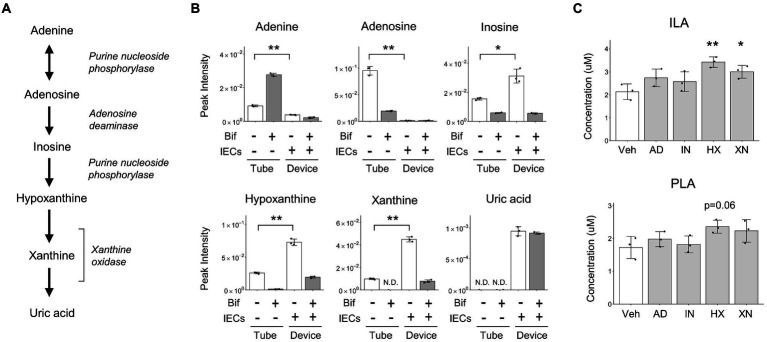
*B. breve* MCC1274 purine metabolism. **(A)** The purine metabolism pathway from adenine to uric acid. **(B)** Peak intensity of purine metabolites detected using CE-FTMS, *p < 0.05, **p < 0.01 (Welch’s *t*-test). **(C)** Aromatic lactic acid concentrations when cultured in the YC medium with purines. *p < 0.05, **p < 0.01 (Dunnett’s test). Data are expressed as the mean ± SD (*n* = 3). AD, Adenosine; HX, Hypoxanthine; IN, Inosine; Veh, vehicle; XN, Xanthine.

**Figure 6:**
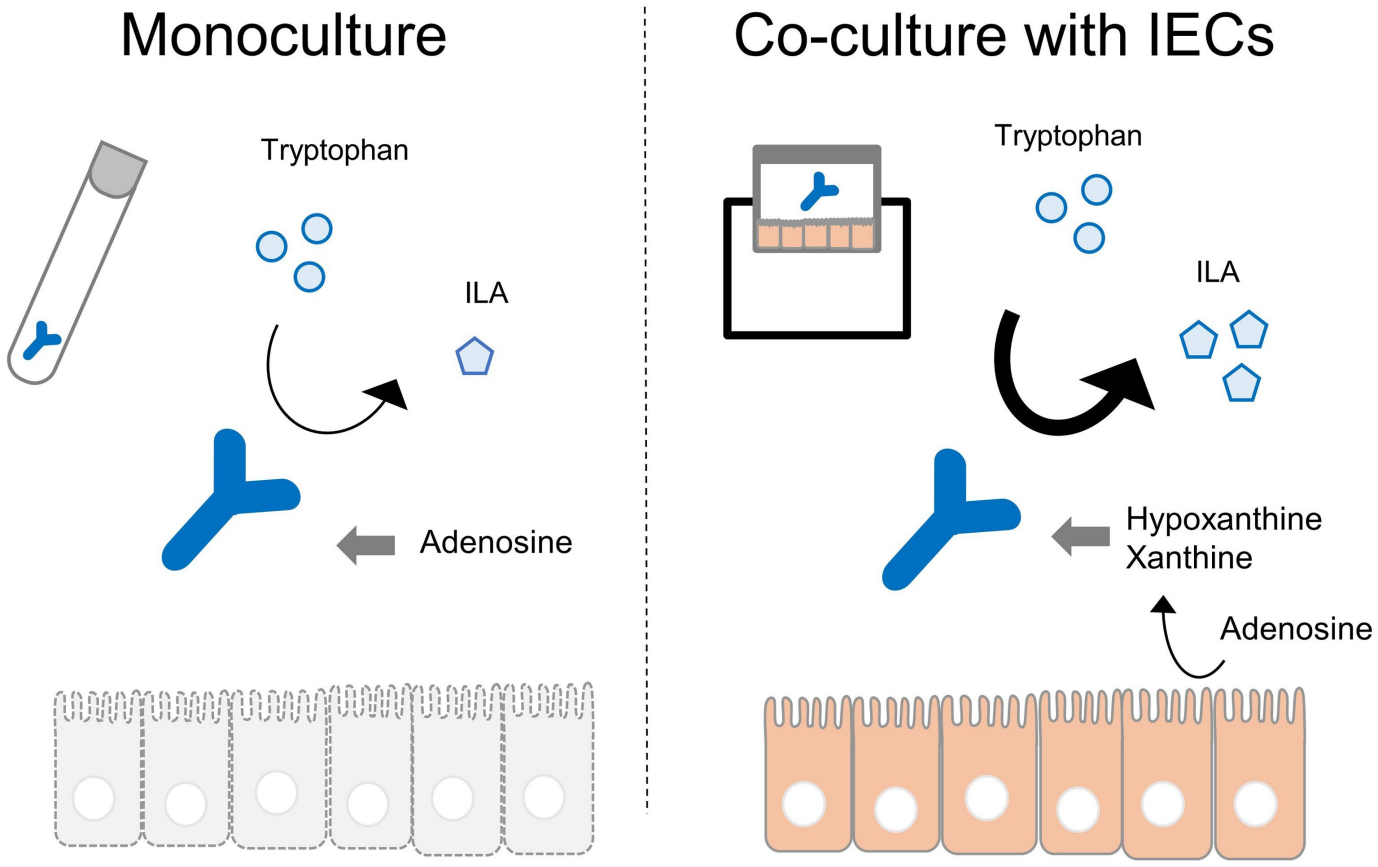
Graphical abstract.

To identify metabolites that enhance the production of ILA in the co-culture conditions, the metabolites with an increased abundance in cluster III of Figure 1C were considered for further analysis. The increase in their abundance in the IECs group and decrease in the co-culture group, suggesting that they were produced by IECs and utilized by *B. breve* MCC1274. Most of these were nucleosides and nucleobases (Figure 1C). On the one hand, in the presence of IECs, the difference in adenosine levels in the medium on the apical side indicated its conversion to uric acid and its intermediate metabolites, such as inosine, hypoxanthine, and xanthine (Figures 5A,B). Corresponding to the conversion, in co-culture conditions, instead of adenosine, *B. breve* MCC1274 consumed inosine, hypoxanthine, and xanthine more. In fact, *in vitro* assays without IECs showed that supplementation with xanthine and hypoxanthine significantly increased *B. breve* MCC1274 ILA production, whereas adenosine supplementation did not cause a significant increase (Figure 5C). These data indicate that the hypoxanthine and xanthine derived from IECs could enhance ILA production by *B. breve* MCC1274 (Figure 6).

## 4. Discussion

In the intestines, IECs interact with microorganisms on the luminal side. However, our knowledge regarding the metabolic interactions between the two is limited due to difficulty in mimicking the gut environment, such as oxygen requirements. To overcome this problem, a Transwell-based static two-layer co-culture system was used in this study. In addition to the two-chamber device, we used F-hiSIEC; iPS-derived small intestinal-like cells, which reflect the small intestine in terms of metabolic activity than well-known intestinal cell lines, such as Caco-2 cell (Onozato et al., 2018). While we did not measure oxygen concentrations in the apical and basolateral sides, with respect to the barrier function, the metabolite compositions were clearly different between the apical and basolateral sides (Supplementary Figures S2A,B). This suggests that the intestinal barrier function was maintained even when the co-culture devise was put under the anaerobic conditions for 48 h.

*B. breve* MCC1274 produces ALAs and their corresponding precursors *in vitro* (Sakurai et al., 2021). To the best of our knowledge, this is the first report of an enhanced production of these metabolites in a co-culture with IECs. Our findings suggest that co-culturing *B. breve* MCC1274 with IECs has a synergistic effect, where the production of ALAs, such as PLA and ILA, is significantly enhanced (Figure 3). PLA and ILA exert immunomodulatory effectors *via* the AhR and hydroxycarboxylic acid receptor 3 (HCA3) (Peters et al., 2019; Sakurai et al., 2019; Meng et al., 2020; Laursen et al., 2021; Sakurai et al., 2021). D-PLA induces pertussis toxin-sensitive migration in human monocytes in an HCA3-dependent manner (Peters et al., 2019). ILA has been reported to attenuate IL-8 production stimulated by interleukin-1β in primary enterocyte derived from necrotizing enterocolitis (NEC) patients and immature human intestinal organoid (Meng et al., 2020). In clinical studies, Laursen et al. reported that ILA reduced IL12p70 production from monocyte stimulated with lipopolysaccharide (Laursen et al., 2021). Another study suggested that ILA up-regulated the gene expression of galectin-1, which suppresses T-cell activation in Th2 and Th17 cells (Henrick et al., 2021). These reports suggest an anti-inflammatory effect of ILA. PLA also has antimicrobial properties (Ning et al., 2017; Sorrentino et al., 2018), and, along with 4-OH PLA, exerts antioxidant activity *via* suppressing ROS production in mitochondria and neutrophils (Beloborodova et al., 2012). Similarly, 2-Hydroxy-4-methylvaleric acid, the concentration of which was also enhanced in the co-culture (Figure 2A), has also been reported as a possible HCA3 ligand with antimicrobial activities against harmful bacteria (Sakurai et al., 2021; Pahalagedara et al., 2022).

In RNA-seq analysis, the upregulated genes were mainly involved in translation and amino acid metabolism pathways, especially amino acid biosynthesis. This suggests that amino acid metabolism by *B. breve* MCC1274 stimulated in the co-culture (Figure 4B). Moreover, guided by metabolomics analysis, of particular interest in this study were the ALA synthesis-related genes. *Bifidobacterium* species that colonize the intestines of breastfed infants have, in addition to human milk oligosaccharide utilization genes, a gene cluster composed of *aat* and *ldh4* (Sakanaka et al., 2019). The former gene presumably encodes aromatic amino acid transaminase that converts aromatic amino acids to aromatic pyruvic acids, while the latter gene encodes lactate dehydrogenase that converts aromatic pyruvic acids into their respective ALAs (Figure 4C; Laursen et al., 2021). In the co-culture, the gene expression of *aat* was significantly increased and that of *ldh4* showed a tendency to increase. These results imply that under the co-culture conditions, the expression of both lactate dehydrogenase and transaminase involved in ALAs production was upregulated, which probably contribute to the conversion of aromatic amino acids into aromatic lactic acids *via* aromatic pyruvic acids. In addition, in the co-culture, there was an enhancement in genes involved in tryptophan and NADH synthesis. Tryptophan is the substrate for ILA synthesis. NADH acts as a cofactor for the dehydrogenase reaction. The data from this study imply that in the co-culture, not only the gene expression directly related to ALA synthesis is enhanced, but so it substrate and cofactor synthesis, which ultimately increased PLA and ILA concentrations.

The relative abundance of purines in the medium was altered in the presence of IECs. Our results suggest that the increase in ILA in the co-culture with IECs was partly due to purine metabolites derived from IECs. Since the enzymes involved in purine metabolism, converting adenosine to uric acid, were highly expressed in the human small intestine (Uhlen et al., 2010) (Human Protein Atlas proteinatlas.org), food-derived adenosine could also convert to inosine, hypoxanthine, and xanthine *in vivo* by IECs.

This indicates that substrates that affect ILA production by *B. breve* MCC1274 are assumed to be more diversified *in vivo*, similar to the results observed in the *in vitro* co-culture system. Some lactic acid bacteria have been reported to utilize extracellular purines for growth (Kilstrup et al., 2005; Martinussen et al., 2010; Yamada et al., 2017). Also, purine nucleoside starvation of *Lactococcus lactis* subsp. *cremoris* could affect its protein expression (Kilstrup et al., 2005). These reports suggest that alteration of purine nucleoside concentrations in the medium could affect the growth and amino acid metabolism of *B. breve* MCC1274, ultimately affecting their ability to synthesize ILA. However, supplementation with xanthine and hypoxanthine had a limited effect on PLA synthesis (Figure 5C). The mechanism underlying the enhancement of ILA production in the *B. breve* MCC1274 *via* supplementation of hypoxanthine and xanthine is yet to be elucidated.

Another possible mechanism related to the enhancement of ALAs production in the co-culture is that IECs and microbes collaborate to synthesize ALAs substrates. Humans possess a gene encoding transaminase for metabolizing phenylalanine and tyrosine to PpyA and HpyA, respectively. In this study, IECs cultured alone produced the same amount of PpyA as *B. breve* MCC1274 cultured alone (Figure 3). Conversely, in co-culture, a significant enhancement in PLA concentration was observed. Thus, the effect of IECs on PpyA synthesis cannot be ruled out. Future studies using *aat* deficient *B. breve* MCC1274 or IECs will determine the main producer of PpyA.

While we attempted to mimic the *in vivo* gut environment to provide relevant results, several limitations remain to be addressed. Firstly, our model lacked some important factors present *in vivo*, such as the microbiota and bile acids present in the small intestinal environment. Furthermore, the *in vitro* three-dimensional structure of IECs, such as the villus, differs from that *in vivo*. Therefore, future studies should use a system that best mimic the *in vivo* environment. Additionally, as indicated by RNA-seq analysis, in the co-culture environment, there was an enhancement in the expression of genes involved in amino acid biosynthesis. However, the observed changes in the gene expression were not directly linked with extracellular metabolic shift in this study. Investigating the intracellular metabolites in *B. breve* MCC1274 will provide insights into this enhancement. With this regard, collection of an appropriate bacteria cell numbers is the most challenging issue when using this co-culture system.

In conclusion, we demonstrated that IECs could affect the metabolism of the probiotic strain *B. breve* MCC1274 using a host-microbe co-culture system. Among the various metabolites, the amounts of the immunomodulatory metabolites PLA and ILA increased in the presence of IECs. However, compared with concentrations in monoculture conditions, the concentrations of aromatic amino acids, which are precursor metabolites of ALAs, were not increased in co-culture conditions. Using the metabolomics analysis data, RNA-seq analysis of *B. breve* MCC1274 was conducted. Our findings revealed an enhancement in the expression of some *B. breve* MCC1274 genes in the presence of IECs. This included amino acid synthesis, cofactor synthesis, and transaminase gene expression. Furthermore, hypoxanthine and xanthine derived from IECs was linked with increased ILA production by *B. breve* MCC1274. These results suggest that IECs-derived factors may enhance the synthesis of beneficial metabolites, such as PLA and ILA, by altering the metabolic activity of *B. breve* MCC1274.

## Data availability statement

The datasets presented in this study can be found in online repositories. The names of the repository/repositories and accession number(s) can be found in the article/Supplementary material.

## Author contributions

AS: conceptualization, investigation, visualization, and writing of the original draft. TN: validation and investigation. SY: supervision. KY: data curation. AG: co-cultivation device development. ToK and TaK: co-cultivation device development and supervision and editing and reviewing manuscript. YY and TH: co-cultivation device development. J-ZX: supervision, editing, and reviewing manuscript. TO: project administration, supervision, editing, and reviewing manuscript. All authors contributed to manuscript revision and read and approved the submitted version.

## Conflict of interest

AS, TN, SY, KY, J-ZX, and TO were employed by Morinaga Milk Industry Co., Ltd. YY and TH were employed by Shimadzu Corp.

The remaining authors declare that the research was conducted in the absence of any commercial or financial relationships that could be construed as a potential conflict of interest.

## Publisher’s note

All claims expressed in this article are solely those of the authors and do not necessarily represent those of their affiliated organizations, or those of the publisher, the editors and the reviewers. Any product that may be evaluated in this article, or claim that may be made by its manufacturer, is not guaranteed or endorsed by the publisher.

## Abbreviations

AAAs: aromatic amino acids
ALAs: aromatic lactic acids
AhR: Aryl hydrocarbon receptor
CE-FTMS: capillary electrophoresis Fourier-transform mass spectrometry
CFU: colony-forming units
DDBJ: DNA Data Bank of Japan
HCA3: hydroxycarboxylic acid receptor 3
DEG: differential gene expression; FC, fold change
HpyA: phenylpyruvic acid
IECs: intestinal epithelial cells
ILA: Indole-3-lactic acid
iPS: induced pluripotent stem cells
IpyA: indole pyruvic acid
KEGG: Kyoto Encyclopedia of Genes and Genomes
LC–MS/MS: liquid chromatography–tandem mass spectrometry
PBS: phosphate-buffered saline
PCA: principal component analysis
PLA: phenyllactic acid
PpyA: phenylpyruvic acid
RNA-seq: RNA sequencing
TOS: Transgalactosylated oligosaccharide
4-OH PLA: 4-hydroxyphenyllactic acid
