# Supplementary figures and images for: Comprehensive analysis of metabolites produced by co-cultivation of Bifidobacterium breve MCC1274 with human iPS-derived intestinal epithelial cells
Source: Front Microbiol. 2023 Apr 13;14:1155438. doi: 10.3389/fmicb.2023.1155438 (PMC10133457; doi:10.3389/fmicb.2023.1155438)

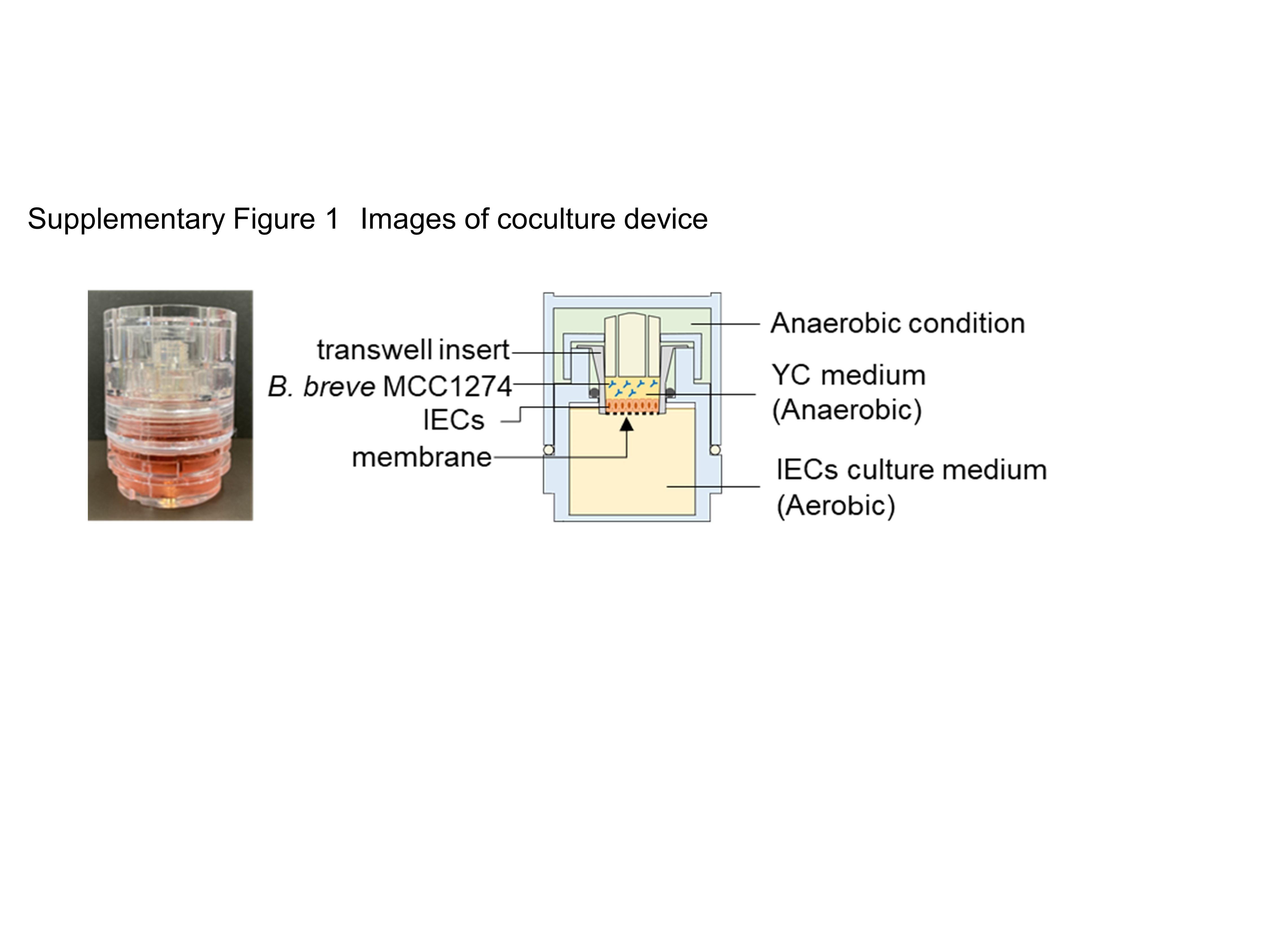

Supplement: Supplementary file 1 [file Image_1.JPEG]

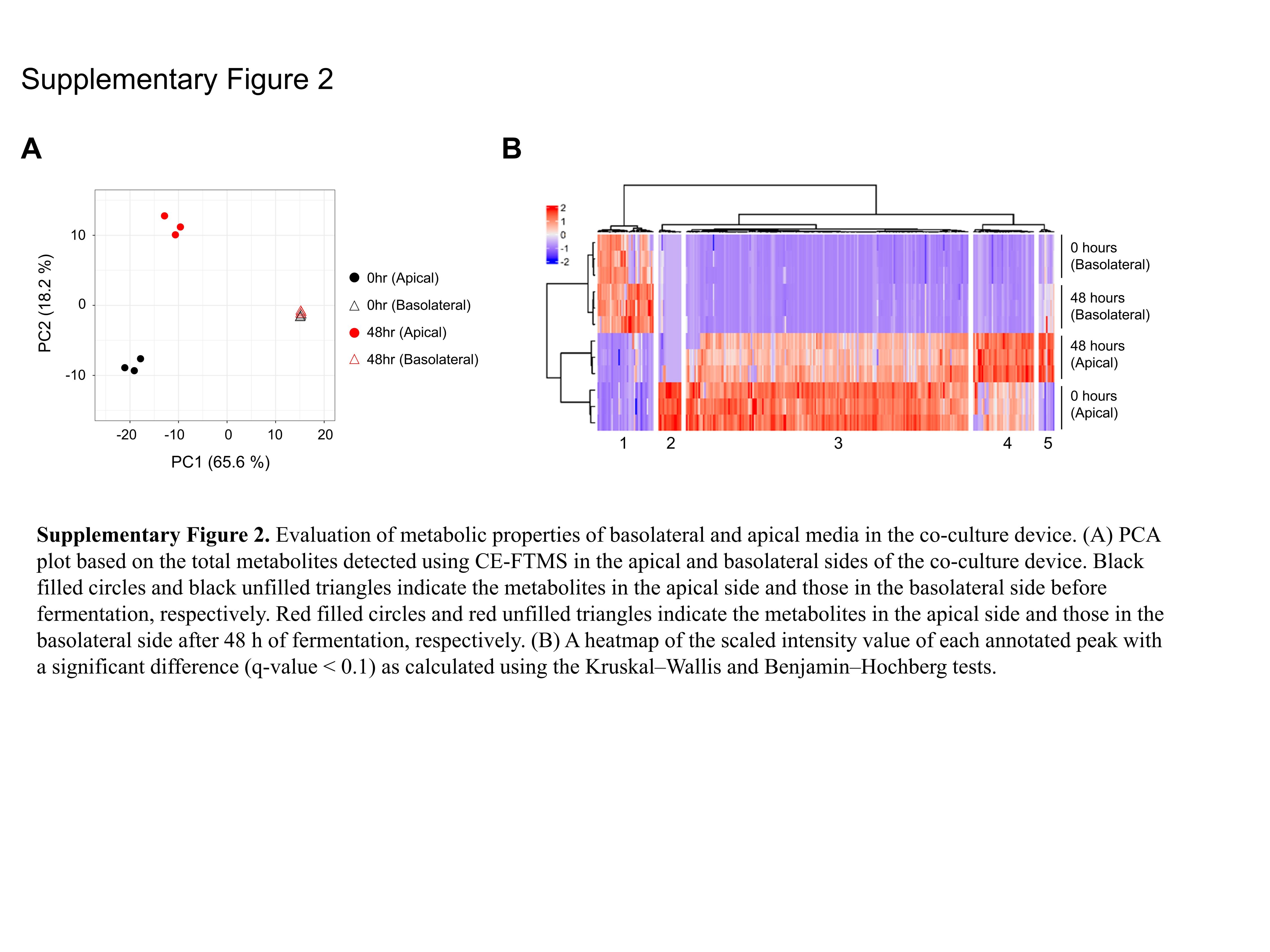

Supplement: Supplementary file 2 [file Image_2.JPEG]
